# Enhanced threat of tick‐borne infections within cities? Assessing public health risks due to ticks in urban green spaces in Helsinki, Finland

**DOI:** 10.1111/zph.12767

**Published:** 2020-09-24

**Authors:** Jani Jukka Sormunen, Niko Kulha, Tero Klemola, Satu Mäkelä, Ella‐Maria Vesilahti, Eero Juhani Vesterinen

**Affiliations:** ^1^ Zoological Museum Biodiversity Unit University of Turku Turku Finland; ^2^ Department of Forest Sciences University of Helsinki Helsinki Finland; ^3^ Marine Research Centre Finnish Environment Institute Helsinki Finland; ^4^ Department of Biology University of Turku Turku Finland; ^5^ Department of Ecology Swedish University of Agricultural Sciences Uppsala Sweden

**Keywords:** infections, lyme disease, parks, recreational, public health, tick bites, ticks

## Abstract

Most tick‐related studies in Europe have been conducted in nonurban areas, but ticks and tick‐borne pathogens also occur in urban green spaces. From a public health perspective, risks regarding tick‐borne infections should be studied in these urban areas, where contacts between infected ticks and humans may be more frequent than elsewhere, due to high human activity. We examined the risk of encountering an infected tick in urban green spaces in Helsinki, Finland. We collected ticks at nine sites throughout Helsinki, recorded the prevalence of several pathogens and identified areas with a high potential for contacts between infected ticks and humans. Moreover, we explored the relationship between the density of *Borrelia burgdorferi* sensu lato‐infected ticks and locally diagnosed cases of borreliosis and compared the potential for human‐tick encounters in Helsinki to those in nonurban areas in south‐western Finland. During 34.8 km of cloth dragging, 2,417 *Ixodes ricinus* were caught (402 adults, 1,399 nymphs and 616 larvae). From analysed nymphs, we found 11 distinct tick‐borne pathogens, with 31.5% of nymphs carrying at least one pathogen. Tick activity was highest in August and September, leading to the density of nymphs infected with *B. burgdorferi* s.l., and concurrently infection risk, to also be highest during this time. Nymph densities varied between the sampling sites, with obvious implications to spatial variation in infection risk. While ticks and tick‐borne pathogens were found in both Helsinki and nonurban areas in south‐western Finland, the estimates of human activity were generally higher in urban green spaces, leading to a higher potential for human‐tick contacts therein. The presence of ticks and tick‐borne pathogens and high local human activity in urban green spaces suggest that they form potential foci regarding the acquisition of tick‐borne infections. Risk areas within cities should be identified and knowledge regarding urban ticks increased.

**Impacts**

- This study shows that there is significant spatial and temporal variations in the risk of getting infected by Lyme disease agents in urban green spaces in Helsinki.
- This study also shows that these urban green spaces generally display a higher potential for human‐tick contacts than more typical, nonurban tick habitats, due to higher human activity.
- The mismatch observed in the current study between borreliosis cases diagnosed in Helsinki and local densities of ticks infected with *Borrelia burgdorferi* sensu lato highlights that disease case data may not precisely reflect local tick situations.

## 1 INTRODUCTION

Ticks and tick‐borne pathogens (TBPs) constitute a growing threat to human well‐being. In Europe, increases in both the abundance of ticks and the numbers of tick‐borne disease (TBD) cases have been reported from many countries during the past few decades (Beaujean et al., 2016; Hofhuis et al., 2016; Jaenson et al., 2012; Sajanti et al., 2017; Vandekerckhove et al., 2019). In Northern Eurasia, climate change has been predicted to extend the latitudinal distribution of ticks and to increase their abundance (Jaenson & Lindgren, 2011; Tälleklint & Jaenson, 1998; Tokarevich et al., 2011). Indeed, observations made during the past few decades indicate that both increases in tick abundance and range expansion have occurred (Bugmyrin et al., 2019; Jaenson et al., 2012; Jaenson et al., 2016; Jore et al., 2011; Laaksonen et al., 2017; Sormunen et al., 2016). In Finland, increasing tick abundance and distribution, and rising numbers of tick‐borne encephalitis (TBE) and borreliosis cases have been reported during the past decade (Laaksonen et al., 2017; Sajanti et al., 2017; Sormunen et al., 2016; Tonteri et al., 2015). Along these broad‐scale changes, increasing tick presence in urban green spaces has also been reported in Finland (Klemola et al., 2019).

Several studies focusing on tick populations in cities have been published (Hansford et al., 2017; Klemola et al., 2019; Kowalec et al., 2017; Maetzel et al., 2005; Oechslin et al., 2017; Rizzoli et al., 2014; Schorn et al., 2011; VanAcker et al., 2019; Žákovská et al., 2008). Overall, these reports suggest that viable tick populations are common in urban green spaces and that the diversity and prevalence of TPBs in these areas are comparable to those measured in nonurban areas. Indeed, while habitats within cities are often spatially restricted and less interconnected than those in more natural areas, suitable host species can nevertheless frequently be found from urban green spaces (Faeth et al., 2011; McKinney, 2008; Rizzoli et al., 2014). Furthermore, human activity is highest in cities, comprising not only of daily activities of residents, but also visiting tourists. Given the recorded presence of ticks and TBPs as well as high densities of humans in cities, contacts between infected ticks and humans may be particularly frequent therein (Rizzoli et al., 2014). However, the rates of contact with infected ticks in urban green spaces remain inadequately quantified.

A tick poses a health risk only if it carries a zoonotic pathogen. Consequently, tick‐borne disease risk has often been measured as the density of ticks infected with a particular pathogen (entomological risk index) (Mather et al., 1996). For *Ixodes ricinus*, nymphs are the major vectors for human infections, as they are more numerous in nature and more difficult to detect when attached to the body than adults, while simultaneously carrying several TBPs at relatively high prevalence (as opposed to larvae). Consequently, density of infected nymphs (DIN) has been used as a key measure for entomological risk regarding *I. ricinus*, similarly to *Ixodes scapularis* in the United States (Diuk‐Wasser et al., 2012; Eisen & Eisen, 2016; Jaenson et al., 2009; Mather et al., 1996; Robertson et al., 2000; Sonenshine & Roe, 2014; Tälleklint & Jaenson, 2014). However, entomological risk does not consider human activity, which is required for human‐tick contact to occur. These contacts, in turn, are the prime requirement for potential risk (DIN) to convert to realized risk (i.e. diseases cases), with areas of high tick and human activity forming potential foci regarding the realization of the risk (Eisen & Eisen, 2016; Sonenshine & Roe, 2014). As such, studying the differences in potential human‐tick contacts between urban green spaces and nonurban areas can help in assessing whether urban green spaces should be focused on in intervention campaigns aiming to reduce tick‐borne diseases (Fischhoff et al., 2019).

For identifying areas where ticks carrying pathogens contact many humans, estimates of human activity are also needed (Eisen & Eisen, 2016; Sonenshine & Roe, 2014). Unfortunately, assessing human activity at specific locations is challenging. In Finland, two different kinds of data are publicly available that may serve as proxies for human activity: national population census data (population density estimated in 1 × 1 km grids; Statistics Finland) and visitor count data (visitor numbers from specific localities, such as national parks and popular outdoors areas). These data have their strengths and weaknesses and cannot be straightforwardly substituted or compared. Visitor count data can provide accurate estimates of human visits to locations that are frequently used for outdoor activities but is only available for certain sites. Population density provides an estimate of the numbers of humans living next to areas inhabited by infected ticks, but the movement patterns or activity of residents cannot be determined from the data (Fischhoff et al., 2019; Kjær et al., 2019). However, these data provide valuable information on potential human activity in areas inhabited by infected ticks. Consequently, observing what they can reveal about the locality‐specific potential for infection risk conversion (from potential to realized risk) is likely a worthwhile pursuit.

In this study, we examine the risk of encountering an infected tick in the urban green spaces of Helsinki, the capital of Finland (population ~650,000 in the city, ~1,170,000 in the metropolitan region). Furthermore, we integrate population census and visitor count data to identify areas with a high potential for (infected) tick‐human contacts. Specifically, we study (a) the occurrence and activity of *I. ricinus* in urban green spaces of Helsinki, (b) the prevalence and diversity of pathogens in collected nymphs, (c) connections between locally diagnosed cases of borreliosis and observed DIN, and (d) differences in the potential for infected tick‐human contacts between urban green spaces of Helsinki and nonurban areas in south‐western Finland.

## 2 MATERIALS AND METHODS

### 2.1 Field collections

**Figure 1:**
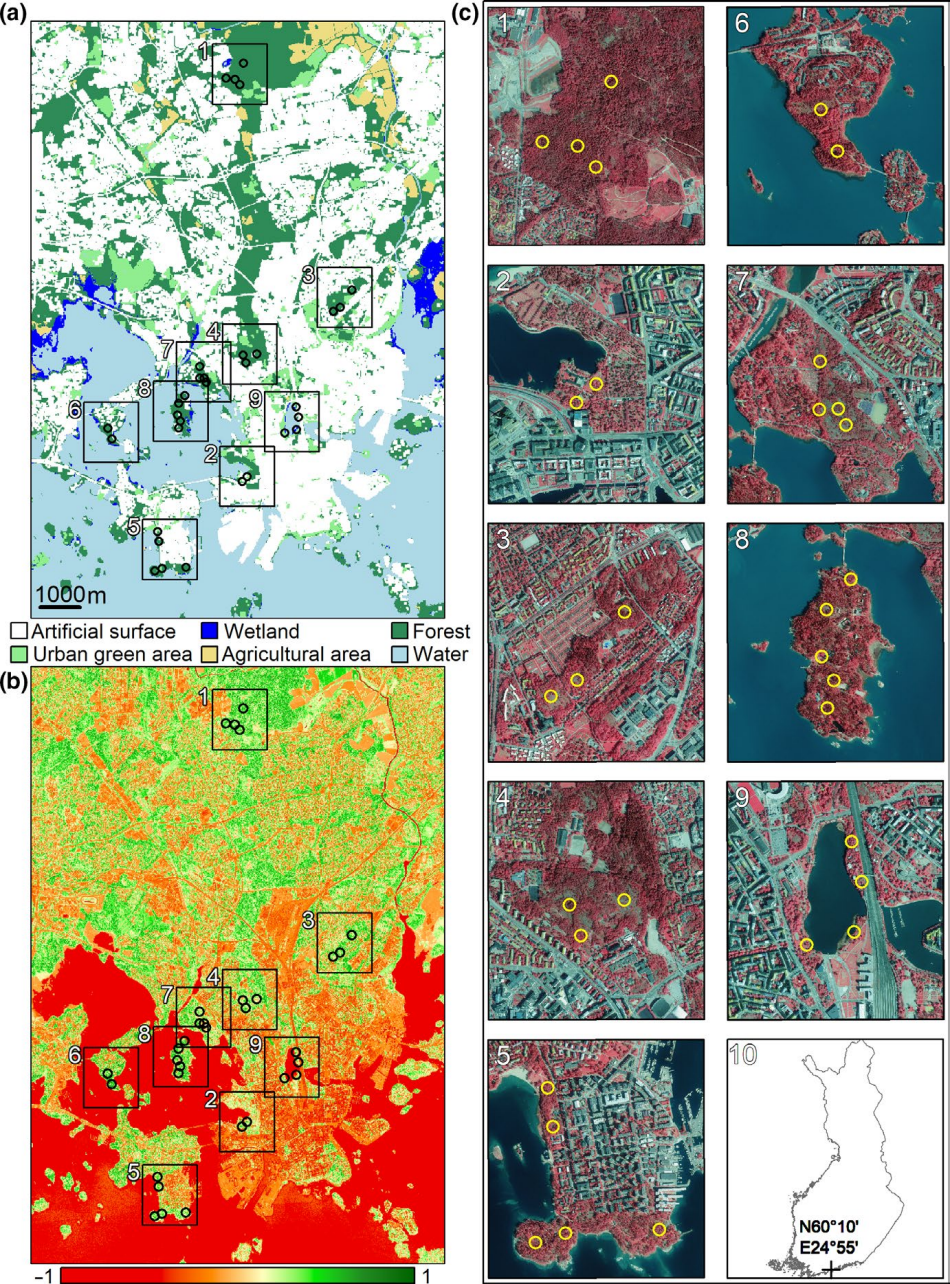
Corine Land Cover (CLC2018; (a) and natural difference vegetation index (NDVI; (b) maps for the Helsinki region, the study sites and sampling locations within the region (c1‐c9), and the location of Helsinki in Finland (c10). The land cover classes are visualized in (a) according to the 1st level CLC2018, with the exception of urban green area (level 3, class 141). The false‐colour aerial photographs visible in panel c were also used to quantify the NDVI. We derived CLC2018 from the Finnish Environment Institute and aerial photographs, taken in 2020, from National Land Survey Finland. Note that the yellow circles in study site maps (c1‐c9) do not represent specific dragging spots, but rather the general areas where dragging was conducted. Sampling locations are c1 Hakuninmaa; c2 Lapinniemi; c3 Kumpula; c4 Laakso; c5 Lauttasaari; c6 Lehtisaari; c7 Meilahti; c8 Seurasaari; and c9 Töölönlahti

Ticks were collected by cloth dragging from May to September in 2019 at nine study sites in Helsinki: Hakuninmaa, Lapinniemi, Kumpula, Laakso, Lauttasaari, Lehtisaari, Meilahti, Seurasaari and Töölönlahti (Figure 1). Land cover classes (Figure 1a), vegetation characteristics (Figure 1b) and landscape configurations (Figure 1c) vary within and between the studied sites. Dragging was conducted once a month at each study site, with 500–1,000 m^2^ dragged in 10 m^2^ subsections each month. The exact distance dragged at each site during any single excursion depended on available time and weather, as well as local tick densities (generally less dragged in high density areas). Dragging spots were chosen separately for each session, based on the operator's assessment of suitable tick microhabitats, and covered varied areas and biotopes within the study sites. In general, patches of coniferous, deciduous or mixed forests were preferred for sampling. The drag consisted of a white 1 m × 1 m linen cloth attached to a wooden pole, with a weight sewn to the posterior end of the cloth. Ticks were collected from the cloth with tweezers after each 10 m drag and stored in ethanol‐filled Eppendorf tubes. The tubes were then delivered to the Zoological Museum at the University of Turku for morphological species identification (Estrada‐Peña et al., 2018b) and laboratory analysis of pathogens.

To facilitate comparisons between the urban green spaces of Helsinki and other, nonurban areas, we calculated DIN for a set of nonurban study sites located in south‐western Finland using previously collected tick density and pathogen prevalence data (Sormunen et al., 2016,2020). As with the study sites in Helsinki, dragging environments at these sites were highly variable, but consisted mainly of coniferous, deciduous or mixed forests. Human inhabitation in the proximity of these sites is very sparse and the proportion of built‐up area low, as opposed to the study sites in the current study. Furthermore, several study sites were located on somewhat remote islands.

As the main focus of field surveys in the current study was to secure tick samples for pathogen analyses and to cover various potential areas of human activity within green spaces for public health risk assessments—rather than to assess the suitability of specific habitats for ticks—dragging was conducted at highly varying locations within study sites. Due to this, the exact locations of drags that caught ticks were not recorded and cannot thus be pinpointed to specific localities/biotopes. A recent study from Germany also reported that climatic variables did not significantly explain differences in tick densities between green spaces, suggesting that host animals and their movements may be more relevant in determining tick presence in urban green spaces (Hauck et al., 2020). Given these considerations and the generally small sizes of the study sites (Figure 1), analyses regarding climatic or landscape variables affecting tick or pathogen presence were not pursued. However, see Technical Appendix for short descriptions of the study sites.

### 2.2 Laboratory analysis

Total DNA and RNA were extracted from 1,386 collected nymphs using NucleoSpin^®^ RNA kits and RNA/DNA buffer sets (Macherey‐Nagel), following the kit protocols (NucleoSpin 96 RNA Core Kit: Rev. 05/April 2014 and RNA/DNA buffer set: Rev. 09/April 2017). RNA extracts were stored at −80°C for later analyses. DNA extracts were stored at −20°C.

DNA samples were screened using real‐time quantitative PCR (henceforth abbreviated qPCR) for bacterial pathogens *Borrelia burgdorferi* sensu lato (henceforth abbreviated BBSL; including separate analyses for *B. afzelii*, *B. garinii*, *B. burgdorferi* s.s. and *B. valaisiana*), *Borrelia miyamotoi*, *Anaplasma phagocytophilum*, *Rickettsia* spp., *Neoehrlichia mikurensis*, *Francisella tularensis* and *Bartonella* spp., and for protozoan parasites *Babesia* spp. Furthermore, RNA samples were screened for tick‐borne encephalitis virus (TBEV). Assay protocols have been reported previously for all screened pathogens (Laaksonen et al., 2018; Sormunen et al., 2018,2020). The primers used for each pathogen are reported in the Technical Appendix, along with further information regarding the analyses.

### 2.3 Assessments of tick risk

For assessing the risk of infection by tick‐borne pathogens (henceforth *infection risk*), density of infected nymphs (DIN) regarding BBSL was calculated for the study sites in Helsinki, as well as for the sites in south‐western Finland. This was done by multiplying site and study month/year‐specific nymph densities with site and study month/year‐specific BBSL prevalence. For study sites in Helsinki, DIN was calculated for each individual 10‐metre drag, and these were then used as independent samples in statistical analyses. Correlations between monthly DIN in Helsinki and estimated tick activity based on data regarding primary Lyme borreliosis (*erythema migrans*) cases from Helsinki were tested by correlation analysis [disease case data from the Register for Primary Health Care Visits, Finnish Institute for Health and Welfare; data described in (Sajanti et al., 2017)].

For estimating site‐specific risk conversion potential (human‐tick contacts), we used two proxies for human activity. Firstly, data regarding estimates of annual visitors to specific study sites were used where available (sources reported in Technical Appendix). Secondly, population estimates were calculated within 500 m and 1 km buffer areas around study sites, using data from a national census (Statistics Finland; population densities in 1 km^2^ grids). Five hundred metres has been suggested as the typical range for daily outdoor activities of local residents (‘neighbourhood’ in Fischhoff et al., 2019). However, we also added estimates from a longer range (1 km), as green spaces within the city are surrounded by highly built‐up areas, potentially leading to longer distances to recreational areas. The potential for human‐tick contacts was calculated by multiplying local DIN with estimates of population density and visitor count data. We use the term *weighted infection risk* to describe the figures attained by this procedure.

### 2.4 Statistical analysis

Study site‐ and month‐specific differences in the numbers of ticks dragged and DIN were modelled by generalized linear mixed models (GLMM), with negative binomial error distribution and log link function. In models of monthly activity and DIN, differences between study sites were controlled for as a random effect (study site), whereas in analysis of study site‐specific activity/DIN, study months were used as a random effect (month). The monthly and study site‐specific probabilities of nymphs being positive for *Borrelia* or *Rickettsia* were modelled by generalized linear mixed models (GLMM) with binary error distribution and logit link function. Study sites and/or months were used as random effects, similarly to the tick activity and DIN analyses above. For other pathogens, too few positive samples were available for analyses.

**Figure 2:**
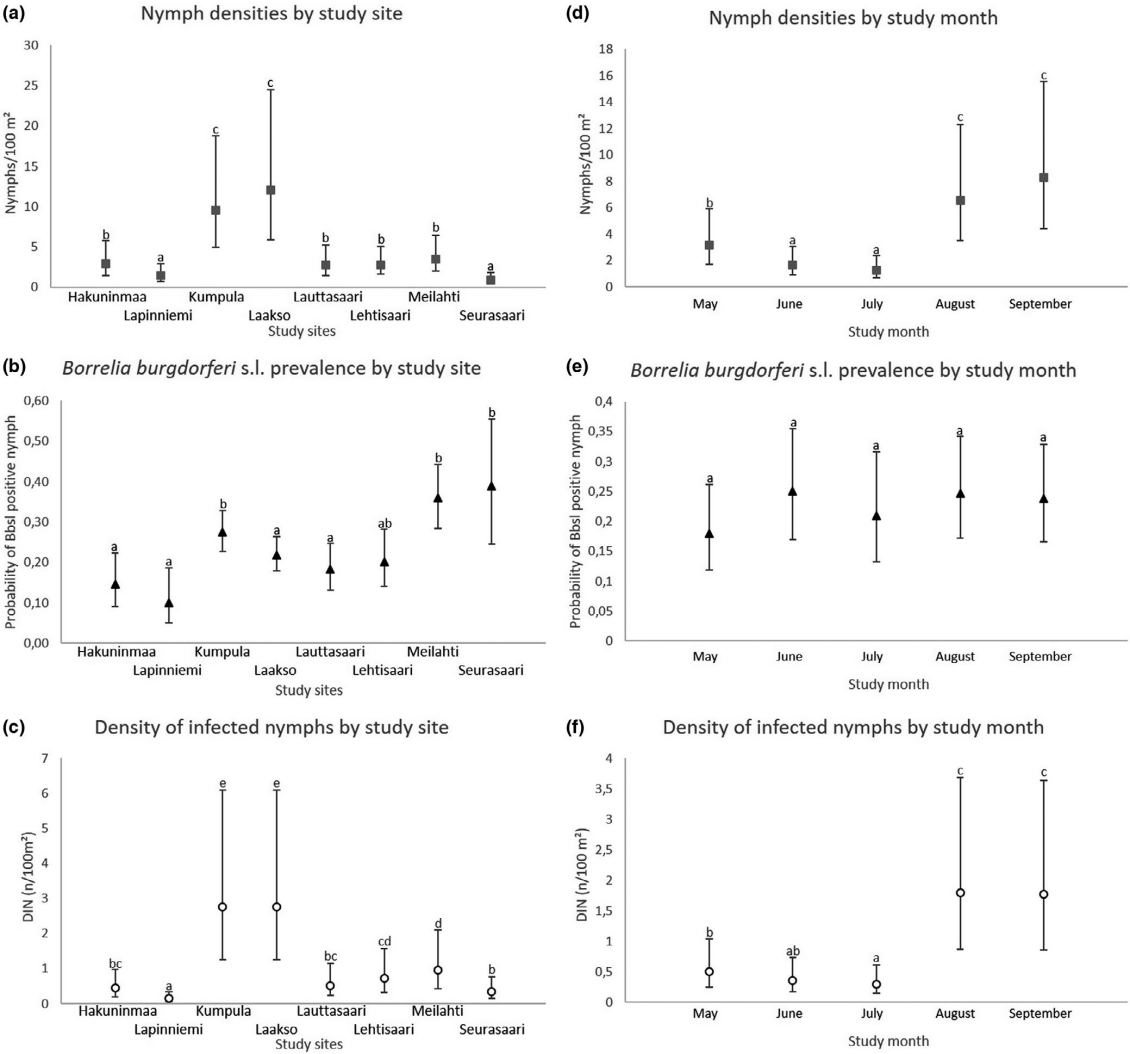
**AQ5:** "AUTHOR: If you would like the figures in your article to appear as colour in print, please promptly post or courier the completed hard copy of the Colour Work Agreement Form (including payment information) to this mailing address: Customer Services (OPI), John Wiley & Sons Ltd, European Distribution Centre, New Era Estate, Oldlands Way, Bognor Regis, West Sussex PO22 9NQ. The form and charge information can be found online at: http://onlinelibrary.wiley.com/journal/10.1111/(ISSN)1863‐2378" Densities and *Borrelia burgdorferi* s.l. prevalence of *Ixodes ricinus* nymphs, and density of infected nymphs (DIN) in Helsinki, by study site (a‐c) or month (d‐e). Mismatching letters denote statistically significant differences between study sites or months with different letters (*p* < .05). Estimated marginal means with 95% confidence intervals are given

**Figure 3:**
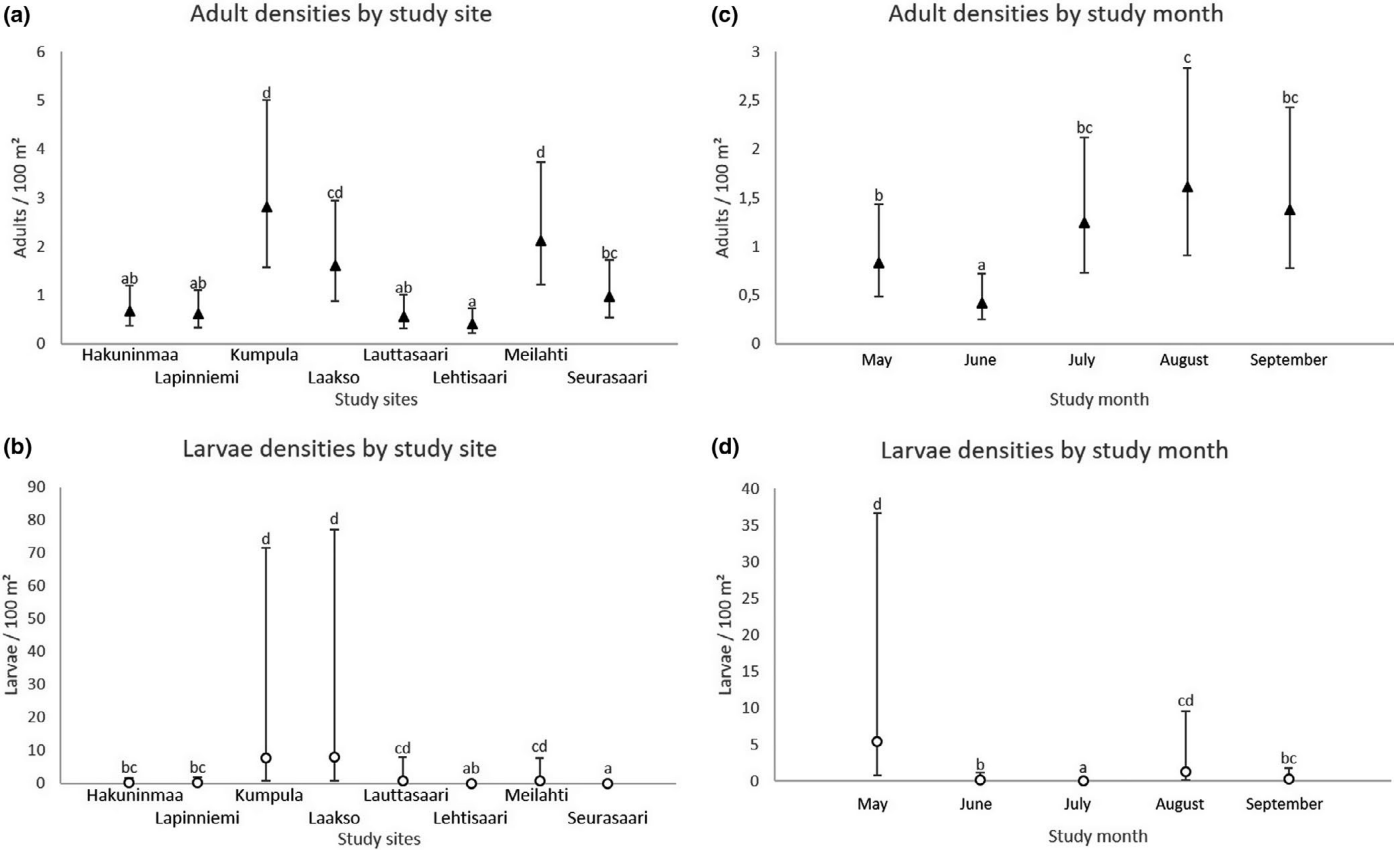
Densities of *Ixodes ricinus* adults and larvae by study site (a‐b) or month (c‐d). Mismatching letters denote statistically significant differences between study sites or months with different letters (*p* < .05). Estimated marginal means with 95% confidence intervals are given

All the GLMMs were run with the GLIMMIX procedure of sas v. 9.4. using maximum likelihood or residual pseudo likelihood estimation. The method by Kenward and Roger (Kenward & Roger, 2009) was chosen to adjust standard errors and denominator degrees‐of‐freedom for tests of the fixed factors. Multiple, a posteriori, pairwise comparisons for differences of the estimated marginal means (i.e. ls‐means in SAS) were adjusted by the Tukey–Kramer method. These results are visually depicted (Figures 2 and 3), using α = 0.05 as a threshold for significant difference.

For estimating whether specific pathogen combinations were present in samples more or less often than expected by random co‐occurrence, Fisher's exact tests were run for each pathogen pair separately. Infection status of samples was analysed as a binomial, categorical variable.

## 3 RESULTS

### 3.1 Tick densities and activity

A total of 2,417 *I. ricinus* (402 adults, 1,399 nymphs and 616 larvae) were caught during 34.8 km of cloth dragging at study sites in Helsinki (Table 1). Ticks were found each month from all study sites, although in varying numbers depending on study site and month (Figures 2 and 3). Overall, peaks in *I. ricinus* activity were observed in May and August–September, with the highest tick densities observed in August and/or September (Figures 2 and 3).

**Table 1:** Dragging lengths, numbers of *Ixodes ricinus* ticks caught and averaged tick densities (±*SE* based on 10 m^2^ subsections as replicates) at study sites in Helsinki

Study site	Distance dragged (metres)	Adults	Nymphs	Larvae	Density/100 m^2^ ± *SE*		
					Adults	Nymphs	Larvae
Hakuninmaa	4,100	32	111	55	0.8 ± 0.1	2.7 ± 0.3	1.3 ± 1.2
Lapinniemi	3,850	26	82	59	0.7 ± 0.1	2.1 ± 0.3	1.5 ± 1.1
Kumpula	3,700	123	308	210	3.3 ± 0.4	8.3 ± 0.6	5.7 ± 1.4
Laakso	3,100	50	416	163	1.6 ± 0.2	13.4 ± 0.9	5.3 ± 0.9
Lauttasaari	4,000	23	170	96	0.6 ± 0.1	4.3 ± 0.5	2.4 ± 1.1
Lehtisaari	4,200	16	124	5	0.4 ± 0.1	3 ± 0.3	0.1 ± 0.1
Meilahti	4,200	86	141	26	2 ± 0.3	3.4 ± 0.3	0.6 ± 0.3
Seurasaari	4,000	37	37	2	0.9 ± 0.2	0.9 ± 0.2	0.1 ± 0.1
Töölönlahti	3,600	9	10	0	0.3 ± 0.1	0.3 ± 0.1	0
TOTAL	34,750	402	1,399	616	1.2 ± 0.1	4 ± 0.2	1.8 ± 0.3

### 3.2 Tick‐borne pathogens

Eleven different pathogens were detected from screened nymphs (*n* = 1,386) (Table 2). Overall BBSL prevalence was 23.0% (95% binomial confidence interval: 20.1%–25.3%) (319/1386), comprising of 193 *B. afzelii* (60.5% of BBSL positive samples), 67 *B. garinii* (21.0%), 36 *B. valaisiana* (11.3%), 35 *B. burgdorferi* s.s. (11.0%) and 11 unidentified *Borrelia* (3.4%). Coinfections with two genospecies were detected in 21 samples, whereas one additional sample was infected with three genospecies. Out of 109 *Rickettsia*‐positive samples (prevalence 7.9 [6.5–9.4]%), 104 were identified as *R. helvetica* (GenBank accession number of reference sample: MH256661.1) and five as *R. monacensis* (KM198341.1). Finally, out of five *Babesia*‐positive samples, three were identified as *B. venatorum* (MH351705.1) and one as *B. capreoli* (KX839234.1), whereas one sample could not be identified due to poor quality of the sequence. No samples positive for *Bartonella*, *F. tularensis* or TBEV were detected. In total, 437 nymphs (31.5 [29.1–34.1]%) were found to carry at least one pathogen.

**Table 2:** Pathogens detected from questing *Ixodes ricinus* nymphs in Helsinki and associated prevalence (%) and its binomial 95% confidence interval

Pathogen	Positive samples	Prevalence	±95 CI
*Borrelia burgdorferi* sensu lato	319	23.0	20.1–25.3
*B. afzelii*	193	14.0	12.2–15.9
*B. garinii*	67	4.8	3.8–6.1
*B. burgdorferi* sensu stricto	35	2.5	1.8–3.5
*B. valaisiana*	36	2.6	1.8–3.6
Unidentified *Borrelia*	11	0.8	0.4–1.4
*Borrelia miyamotoi*	10	0.7	0.4–1.3
*Rickettsia*	109	7.9	6.5–9.4
*R. helvetica*	104	7.5	6.2–9.0
*R. monacensis*	5	0.4	0.1–0.8
*Babesia*	5	0.4	0.1–0.8
*B. venatorum*	3	0.2	0.0–0.6
*B. capreoli*	1	0.1	0.0–0.4
*Neoehrlichia mikurensis*	34	2.5	1.7–3.4
*Anaplasma phagocytophilum*	15	1.1	0.6–1.8
Tick‐borne encephalitis virus	0	0	‐
Any pathogen	437	31.5	29.1–34.1

No<del author="Jani Jukka Sormunen" command="Delete" timestamp="1600253546697" title="Deleted by Jani Jukka Sormunen on 9/16/2020, 1:52:26 PM" class="reU3">te</del>

Number of analysed samples for each pathogen was 1,386. Note that the number of *Borrelia burgdorferi* s.l. positive samples differs from the sum of all genospecies and unidentified *Borrelia* due to coinfections.

A total of 70 nymphs (5.1 [4.0–6.3]%) were detected to be carrying two or more pathogens (see Technical Appendix for a full listing). Among these, several combinations of pathogens were observed to be more or less frequent than expected by random co‐occurrence. Those observed to be more common than expected were *B. afzelii* and *N. mikurensis* (4.4 expected, 14 observed; Fisher's exact test [FET], *p* < .0001), *B. afzelii and Rickettsia* (14.2 expected, 22 observed; FET, *p* = .02), *B. afzelii and B. burgdorferi* s.s. (4.6 expected, 10 observed; FET, *p* = .008), and *B. garinii* and *B. valaisiana* (1.3 expected, 4 observed; FET, *p* = .03). *Borrelia afzelii* and *B. valaisiana* were the only combination observed less often than expected (4.6 expected, 0 observed; FET, *p* = .03) (*n* = 1,386 for all analyses). Relatively high proportions of all positive identifications of *B. burgdorferi* s.s., *N. mikurensis* and *Rickettsia* were coinfections with *B. afzelii* (28.6, 41.2, and 20.2%, respectively).

Nymph BBSL prevalence varied among study sites (Figure 2) (GLMM, *n* = 1,376, *F*_8, 1,367_ = 4.37, *p* < .0001), but no significant differences were detected between study months (GLMM, *n* = 1,376, *F*_4, 1,371_ = 1.09, *p* = .36). Regarding *Rickettsia*, no statistical differences were detected between study sites or months (GLMM, *n* = 1,376, *F*_8, 1,367_ = 1.74, *p* = .08; GLMM, *n* = 1,376, *F*_4, 1,371_ = 1.22, *p* = .30).

### 3.3 Density of infected nymphs and tick risk

**Figure 4:**
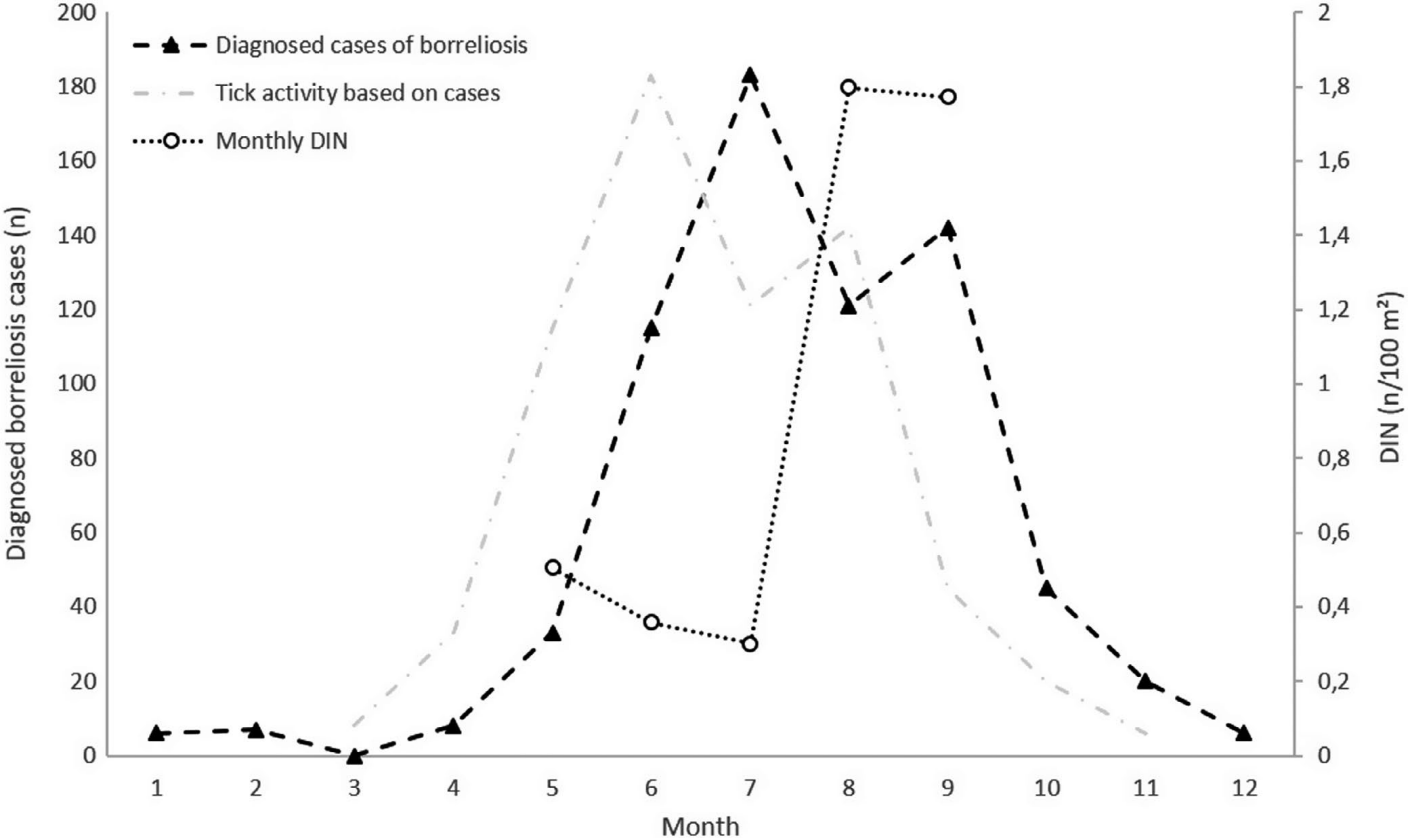
Observed monthly densities of *Ixodes ricinus* nymphs infected with *Borrelia burgdorferi* s.l. (DIN; averaged for Helsinki green spaces) and primary borreliosis (*erythema migrans*) cases diagnosed in Helsinki in 2019. The grey line represents predicted tick activity patterns based on diagnosed cases, estimated with a 1‐month delay from tick bite to diagnosis. Note the different scales on *y*‐axes

DIN was found to vary between study months, with September having the highest DIN, and June and July the lowest (GLMM, *n* = 3,385, *F*_4, 3,373_ = 55.26, *p* < .0001) (Figure 2). DIN also varied across study sites (GLMM, *n* = 3,385, *F*_7, 3,373_ = 52.13, *p* < .0001) (Figure 2). Nymph density was highly correlated with DIN (*n* = 3,385, *r* = 0.92, *p* < .0001) and explained most its variance (*R^2^* = 0.85), whereas corresponding figures were a lot lower regarding BBSL prevalence (*n* = 3,385, *r* = 0.17, *p* < .0001, *R^2^* = 0.03). There was a negative trend between observed DIN and predicted tick activity based on borreliosis cases diagnosed in the Helsinki healthcare district (estimated with a 1 month delay from tick bite to diagnosis), but due to low samples size, there was no power to detect it statistically (*n* = 5 months, *r *= −0.51, *p* = .38) (Figure 4).

Estimates of weighted infection risk varied by up to orders of magnitude across green spaces in Helsinki and study sites in south‐western Finland (Table 3). Three green spaces in Helsinki and the botanical garden in Ruissalo, near Turku, displayed the highest potential for contacts between infected ticks and humans based on visitor count data, followed by two rural islands with high DIN but lower visitor counts, Boskär and Seili. Kumpula and Laakso were revealed as particularly high‐risk areas due to high amounts of people living in the vicinity (Table 3). Overall, highly varying estimates for human activity were obtained from the two data sets.

**Table 3:** Densities of *Ixodes ricinus* nymphs and nymphs infected with *Borrelia burgdorferi* s.l. (DIN), and weighted infection risk estimates based on visitor count and population density data for study sites in south‐western Finland (A) and green spaces in the city of Helsinki (B). WGS84 coordinates are given for study sites outside of Helsinki; for sites in Helsinki, see Figure 1

Study area	Coordinates	Nymphs/100 m^2^	DIN (/100 m^2^) (year)	Visitor count (year)^a^	Weighted infection risk	Population density within (census year 2015)		Weighted infection risk based on population density	
						500 m	1 km	500 m	1 km
A. South‐western Finland									
Pähkinäinen	60°19′42.0″N 21°41′17.9″E	9.1	1.8 (2013)	2,062 (2013)	3.7 × 10^3^	0	0	0	0
Vepsä	60°22′23.2″N 22°04′37.6″E	0.7	0 (2013)	10,000 (2013)	0	0	0	0	0
Maisaari	60°19′45.3″N 21°53′50.2″E	1.7	0.3 (2013)	2,600 (2013)	7.8 × 10^2^	0	1	0	0.3
Boskär	60°02′02.0″N 21°46′13.8″E	34.2	10.6 (2013)	1,300 (2014)	1.3 × 10^4^	0	0	0	0
Berghamn	60°03′01.5″N 21°47′59.4″E	7.75	0.04 (2013)	3,000 (2014)	1.2 × 10^2^	4	5	0.2	0.2
Seili	60°14′19.6″N 21°57′37.5″E	24	5.8 (2017)	15,000 (2017)	8.7 × 10^4^	0	2	0	12
Ruissalo B.G.^b^	60°26′00.3″N 22°10′24.1″E	9.9	3.6 (2019)	104,000 (2019)	3.7 × 10^5^	13	42	47	1.5 × 10^2^
Askainen	60°34′44.2″N 21°49′06.3″E	2.3	0.3 (2014)	‐	‐	0	2	0	0.6
Rihtniemi	61°03′58.4″N 21°19′16.5″E	2.3	0.7 (2014)	‐	‐	29	29	20	20
Tvärminne	59°50′31.6″N 23°12′06.3″E	8.6	1 (2018)	‐	‐	8	8	8	8
B. Helsinki									
Hakuninmaa		2.7	0.4 (2019)	‐	‐	1,792	2,654	7.2 × 10^2^	1.1 × 10^3^
Lapinniemi		2.1	0.2 (2019)	‐	‐	9,838	22,634	2 × 10^3^	4.5 × 10^3^
Kumpula		8.3	2.3 (2019)	‐	‐	4,363	11,139	1 × 10^4^	2.6 × 10^4^
Laakso		13.4	3 (2019)	‐	‐	11,757	15,640	3.5 × 10^4^	4.7 × 10^4^
Lauttasaari		4.3	0.9 (2019)	208,000 (1995)	1.9 × 10^5^	3,808	8,586	3.4 × 10^3^	7.7 × 10^3^
Lehtisaari		3	0.6 (2019)	10,000 (1995)	6 × 10^3^	1,344	1,838	8.1 × 10^2^	1.1 × 10^3^
Meilahti		3.4	1.2 (2019)	700,000 (1995)	8.4 × 10^5^	4,226	7,099	5.1 × 10^3^	8.5 × 10^3^
Seurasaari		0.9	0.4 (2019)	700,000 (2019)	2.8 × 10^5^	0	1,122	0	4.5 × 10^2^

^a^ Visitor count data sources reported in the Technical Appendix.

^b^ Ruissalo Botanical Garden.

## 4 DISCUSSION

In the current study, we show that (a) ticks and tick‐borne pathogens are common in urban green spaces in Helsinki, the capital of Finland, (b) the density of infected ticks (DIN) varies depending on study location and sampling time and is mostly dependent on local tick densities and (c) while the estimates of human activity weighted infection risk are variable, they are generally higher in urban green spaces of Helsinki than in the studied nonurban sites located in south‐western Finland. Finally, we show that TBP diversity is high within the city and that rodent‐associated pathogens are more common than bird‐associated ones and report *Babesia capreoli* for the first time from ticks in Finland.

### 4.1 Tick densities and activity

Ticks were found in varying numbers from urban green spaces in Helsinki. In addition to more forested areas, ticks were also found from small patches of suitable habitat, including patches of trees in the middle of maintained grass lawns. While not quantified here, the presence of leaf litter was determined to be a major factor predicting tick occurrence by field workers, as suggested previously (Dautel & Kahl, 1999). The presence and abundance of all tick life stages in urban green spaces suggests that they are able to carry out their life cycles and form breeding populations even in these varied and potentially spatially restricted habitats (Dautel & Kahl, 1999; Rizzoli et al., 2014).

Peaks in the activity of *I. ricinus* nymphs were observed in May and in August–September, with the highest densities occurring in August–September. A similar trend was observed for adults, but with only June noticeably differing from other months with its low activity. For larvae, variation between months was particularly high, likely due to the aggregated nature of their occurrence. As such, the estimates of larval activity should be interpreted with caution. In general, these patterns of activity are well in line with those commonly reported for *I. ricinus* and are liable to change based on differences in environmental factors across years and study areas (Gray, 1991; Sirotkin & Korenberg, 2018). Indeed, it should be noted that these assessments of tick activity are based on data from a single year and a limited number of excursions to each study site. The activity and density of ticks may vary across years at any specific site, affecting also the associated public health risks. However, in the current study, the general seasonal trends of nymph activity, and consequently the measured public health risk, were similar across all study sites (Figure 2d). As such, similar trends may be expected to have occurred in other green spaces in the city as well.

### 4.2 Tick‐borne pathogens (TBPs)

The findings of the current study regarding TBPs concur with previously reported results, suggesting that TBP prevalence and diversity in urban green spaces are comparable to those in nonurban areas (Kowalec et al., 2017; Maetzel et al., 2005; Oechslin et al., 2017; Rizzoli et al., 2014). Bacteria from the BBSL group were expectedly the most common pathogens detected. Results from all over Europe have shown that BBSL infection is common in *I. ricinus*, although prevalence is spatially varying (Estrada‐Peña et al., 2018a; Strnad et al., 2017). Regarding BBSL genospecies, as was recently observed also in Turku, Finland (Klemola et al., 2019), rodent‐associated genospecies were more common than bird‐associated ones, suggesting that rodents are more common hosts for larvae than birds in these environments. Similar observations regarding genospecies composition have been reported from many other urban areas around Europe as well (Kowalec et al., 2017; Maetzel et al., 2005; Oechslin et al., 2017; Rizzoli et al., 2014). In addition to *B. afzelii* and *B. burgdorferi* s.s., also rodent‐associated *N. mikurensis* was detected from samples, frequently together with *B. afzelii*. As only nymphs were analysed, it may be expected that the majority of pathogens detected were obtained during the larval blood meal from a single host (or from co‐feeding ticks during this meal), although the possibility for interrupted and re‐instated feeding also exists (Randolph et al., 1996; Richter et al., 2012; Voordouw, 2015). However, for the precise identification of pathogen sources (i.e. source species), general host–pathogen associations are no longer sufficient, and specific data on tick blood meal sources are required. Indeed, the development of reliable and cost‐effective methods for identifying blood meal sources from host‐seeking ticks should be pursued, in order to reveal (localized) host–pathogen associations and help determine the enzootic cycles of TBPs and variation therein.

A single tick carrying *Babesia capreoli* was found from Hakuninmaa, forming the first report of this pathogen from Finland. The species has previously been reported from ticks and/or roe deer in neighbouring Sweden and Norway (Andersson et al., 2016; Øines et al., 2012), so its occurrence also in Finland is not surprising. Incidentally, Hakuninmaa, located in the central park of Helsinki, is one of the few study sites in the current study that house roe deer (*Capreolus capreolus*) and white‐tailed deer (*Odocoileus virginianus*) populations, which are suspected to be reservoir hosts for the pathogen (Malandrin et al., 2010).

Simultaneous infections with two or more TBPs have on occasion been demonstrated to lead to more severe diseases in humans (Swanson et al., 2006). Thus, it is important to observe naturally occurring coinfections in ticks. In the current study, various combinations of coinfections were detected in analysed nymphs, with the general coinfection prevalence being somewhat higher than commonly observed for nymphs (typically <5%, but with higher values also reported) (Klitgaard et al., 2019; Lommano et al., 2012; Nieto & Foley, 2009; Overzier et al., 2013; Wójcik‐Fatla et al., 2009). However, the figures reported are difficult to compare, as different combinations of pathogens have been screened in different studies. The positive and negative associations between co‐occurring pathogens detected in the current study were logical: coinfections involving mammal/rodent‐associated pathogens (*B. afzelii*, *B. burgdorferi* s.s., *N. mikurensis*) and bird‐associated pathogens (*B. garinii*, *B. valaisiana*) were more common than expected by random co‐occurrence, whereas co‐occurrence of pathogens associated with different reservoir animal groups was less common than expected.

### 4.3 Density of infected nymphs and weighted infection risk

Density of infected nymphs (DIN) was found to vary among both study sites and months. In general, tick densities were the most important factor in determining DIN. Consequently, it would appear that while the presence of BBSL is required for infected ticks to exist, the variation caused by differences in its prevalence is minimal compared with that caused by changes in tick abundance, as suggested previously (Tälleklint & Jaenson, 2014). Consequently, BBSL prevalence alone should not be used for risk assessments, whereas tick densities alone might suffice (Tälleklint & Jaenson, 2014). However, the optimal situation would always be to obtain DIN values (Eisen & Eisen, 2016).

Interestingly, the higher DIN in urban green spaces in August–September is not reflected in cases of primary borreliosis diagnosed within the city. In fact, the association between observed DIN and predicted tick activity based on patient cases (estimated with a 1‐month delay from tick bite to diagnosis) appeared to be negative. As DIN is expected to depict the potential risk of contracting borreliosis from ticks (Mather et al., 1996), one would expect a high amount of infections being contracted at times of high DIN—with the base assumption that human activity is roughly consistent. However, human outdoor activity might not be consistent from May to September. Furthermore, there is likely a difference between the risk associated with outdoor activities commonly undertaken in cities (jogging, walking the dog) and nonurban areas (hiking, mushroom and berry picking, hunting), leading to different risk conversion rates (Randolph, 2010). All in all, it would appear that an unknown proportion of borreliosis cases diagnosed in Helsinki likely represent infections obtained elsewhere, making it difficult to assess trends within the city based on disease case data. In any case, this mismatch between locally observed DIN and borreliosis cases highlights the need for further knowledge regarding the specific circumstances leading to human‐tick contacts, as well the search for other predictor variables besides DIN that encompass human behaviour and land use (Eisen & Eisen, 2016; Sen et al., 2015; Vanwambeke et al., 2010).

Highly varying estimates for human activity were obtained from the two proxy data sets (population census and visitor count data). While visitor count data likely more accurately predict the frequency of risk area visits, it is greatly hampered by its availability. Hence, the use of population densities obtained by national censuses seems like a more applicable option for mapping risk areas, particularly for areas with high population density. For example, such data could be used for identifying high‐risk areas particularly within cities by (a) first mapping out the estimated population densities adjacent to different urban green spaces, (b) assessing whether habitats in green spaces surrounded by the highest densities of humans appear suitable for tick inhabitation and (c) obtaining DIN values from suitable green spaces by capturing and analysing ticks. However, estimations based on population densities may fail to identify popular recreational areas within cities, as demonstrated in the current study by the mismatch of visitor count and population density data from the Seurasaari study site. Instead, various user‐generated observations such as mobile phone and/or social media data may provide a useful alternative for determining the usage of urban green spaces and, consequently, for identifying high‐risk areas (Heikinheimo et al., 2020). Nevertheless, the more people living close to a risk area there are, the more people may be expected to enter them. Therefore, incorporating even approximate estimates of human activity to risk assessments via population census data may provide additional insight on locality‐specific frequencies of human‐tick contacts and consequently help in identifying areas where interventions are most needed (Fischhoff et al., 2019).

## CONFLICT OF INTEREST

The authors have no conflict of interest to declare.

## ETHICAL APPROVAL

No human or animal subjects were involved in this study.

## APPENDIX 1

### Technical Appendix To:

Enhanced threat of tick‐borne infections within cities? Assessing public health risks due to ticks in urban green spaces in Helsinki, Finland

Jani Jukka Sormunen, Niko Kulha, Tero Klemola, Satu Mäkelä, Ella‐Maria Vesilahti, and Eero Juhani Vesterinen

Text A1: Descriptions of study sites

Text A2: Additional methods

Table A1. Primers and probes used in tick‐borne pathogen screening and sequencing

Table A2. Detected co‐infections in host‐seeking nymphs from Helsinki

Table A3. Results from the statistical analyses regarding tick densities

Table A4: Visitor count data sources

Supplemental references

### Text A1: Descriptions of study sites

Hakuninmaa: Mixed forest, dominated by conifers (*Picea abies* and *Pinus sylvestris*). This area is commonly used for outdoor activities, being a part of the Helsinki central park, which has an estimated 2,000,000 visitors annually. Consequently, plenty of tracks and trails can be found and are used by citizens across the forest. Hakuninmaa is the largest and most forested of the study sites, and the central park houses white‐tailed deer (*Odocoileus virginianus*) and roe deer (*Capreolus capreolus*) populations, which are mostly missing from other study sites.

Lapinniemi: A popular public park, consisting mainly on maintained lawn areas. However, the lawn is speckled with tree stands with leaf litter and understory vegetation, and some meadow areas, where dragging was conducted. Both deciduous and coniferous trees are present, with deciduous tree stands dominating. The area contains no forests.

Kumpula: A small mixed forest enclosed by built‐up area. Collections were mostly focused in areas dominated by deciduous trees, where ample leaf litter was present. A nearby lido and community garden draw citizens to this area. Likewise, the community garden draws in, for example, European hares (*Lepus europaeus*).

Laakso: Mixed forest, with both conifer and deciduous tree dominated areas. Part of the Helsinki central park like Hakuninmaa, forming the southernmost edge of the more‐or‐less continuous central forest. As with Hakuninmaa, plenty of tracks and forest trails crisscross forests at Laakso.

Lauttasaari: Mixed forest, with truly mixed areas as well as areas dominated by either conifers or deciduous trees. Another popular outdoors area.

Lehtisaari: A small forested area on an urban island. Mixed forests, but dragging mostly conducted in parts dominated by deciduous trees. As with most forested areas in the capital region, plenty of tracks and trails used by citizens can be found in the forest.

Meilahti: A rather small mixed forest area in the middle of built‐up areas, surrounded by sporting facilities and a botanical garden. Plenty of open (often bare‐rock) and dry areas, with shadier and more humid areas occasionally found. Known to house red foxes (*Vulpes vulpes*).

Seurasaari: A forested island. An extremely popular outdoors area locally, visited by an estimated 700,000 people in 2019. The northern part of the island contains an outdoor museum. The forests are mixed, but mostly dominated by conifers (mainly *P. abies*). Many local school classes visit the island for excursions, as well as thousands of tourists. It is also well known for its tame squirrels and birds, which are often photographed there.

Töölönlahti: An extremely popular public park in the middle of Helsinki, used for recreational activities and commuting. The western part of the park is mostly well‐maintained lawns, whereas the eastern parts have areas with leaf litter and longer vegetation in general. No continuous canopy cover, but tree stands formed mostly of deciduous trees are present.

### Text A2: Additional methods

Real‐time quantitative PCR (henceforth abbreviated qPCR) assays were carried out using SensiFAST^™^ Probe Lo‐ROX Kit (for DNA) and SensiFAST^™^ Probe Lo‐ROX One‐Step Kit (for RNA) (Bioline, Germany). All DNA/RNA samples were analysed in two replicate reactions carried out on 96 or 384‐well plates. Analyses regarding *Borrelia* were carried out on individual DNA samples. For the screening of all other pathogens, samples were analysed in pools (12 samples per pool, 5 μl of each sample) due to low expected prevalence. Individual samples from a pool found positive were subsequently re‐analysed separately. At least two non‐template controls (template replaced with distilled water) were used in each assay. Samples were considered positive when successful amplification was detected in both replicate reactions or in two consecutive assays. Assay protocols have been reported previously for all screened pathogens (Laaksonen et al., 2018; Sormunen et al., 2018). The primers used for each pathogen are reported in Table A1

Samples found positive for *Rickettsia* or *Babesia* with qPCR were subsequently amplified by conventional PCR and Sanger‐sequenced in order to determine species (Table A1). Likewise, BBSL‐positive samples that could not be identified to the genospecies level by qPCR were Sanger‐sequenced (Table A1). Assay protocols and mastermix contents for PCR amplification of these were as reported previously (Sormunen et al., 2018; 2016).

**Table A1:** Primers and probes used in tick‐borne pathogen screening and sequencing

Oligo name	Primer/probe target	Sequence 5′→3′	Reference
qPCR:			
Bb23Sf	*B. burgdorferi* 23S RNA	CGAGTCTTAAAAGGGCGATTTAGT	Courtney et al., 2004
Bb23Sr	*B. burgdorferi* 23S RNA	GCTTCAGCCTGGCCATAAATAG	
Bb23Sp	*B. burgdorferi* 23S RNA	[FAM]‐AGATGTGGTAGACCCGAAGCCGAGTG‐[BHQ1]	
Baf‐RecA‐F	*B. afzelii recA*	AGTCAGCCTGATACCGGAGA	Klemola et al., 2019
Baf‐RecA‐R	*B. afzelii recA*	ATTTTGGGGTCAAAGCTGCC	
Baf‐RecA‐P	*B. afzelii recA*	[FAM]‐TGCCGAACATTTAATTAGAAG‐[BHQ1]	Tveten, 2013
Bga‐RecA‐F	*B. garinii recA*	ATGCAAAAGCTTTGGGGGTT	Klemola et al., 2019
Bga‐RecA‐R	*B. garinii recA*	AGGGGTTAAAGCTGCTACAGA	
Bga‐RecA‐P	*B. garinii recA*	[HEX]‐TTGCCGAACATTTAATCAGAA‐[BHQ1]	Tveten, 2013
Bbss‐RecA‐F	*B. burgdorferi* s.s. *recA*	CCTGATACCGGAGAGCAAGC	Klemola et al., 2019
Bbss‐RecA‐R	*B. burgdorferi* s.s. *recA*	GGGGTTAAAGCCGCTACAGA	
Bbss‐RecA‐P	*B. burgdorferi* s.s. *recA*	[HEX]‐TTGCTGAGCATTTAATCAGAA‐[BHQ1]	Tveten, 2013
Bva‐RecA‐F	*B. valaisiana recA*	TGGTCCTGAGTCGTCTGGTA	Klemola et al., 2019
Bva‐RecA‐R	*B. valaisiana recA*	CTTGCTCTCCGGTGTCAGG	
Bva‐RecA‐P	*B. valaisiana recA*	[Cy5]‐AGGTTCAAAAAGAAGGTGGTAT‐[BHQ2]	Tveten, 2013
Bmi‐F	*B. miyamotoi glpQ*	CACGACCCAGAAATTGACACA	Vayssier‐Taussat et al., 2013
Bmi‐R	*B. miyamotoi glpQ*	GTGTGAAGTCAGTGGCGTAAT	
Bmi‐P	*B. miyamotoi glpQ*	[FAM]‐TCGTCCGTTTTCTCTAGCTCGATTGGG‐[BHQ1]	
Bart‐ssRA‐F	*Bartonella ssRa*	GCTATGGTAATAAATGGACAATGAAATAA	Diaz et al., 2012
Bart‐ssRA‐R	*Bartonella ssRa*	GCTTCTGTTGCCAGGTG	
Bart‐ssRA‐P	*Bartonella ssRa*	[FAM]‐ACCCCGCTTAAACCTGCGACG‐[BHQ1]	
Rspp‐F	*Rickettsia gltA*	GAGAGAAAATTATATCCAAATGTTGAT	Labruna et al., 2004
Rspp‐R	*Rickettsia gltA*	AGGGTCTTCGTGCATTTCTT	
Rspp‐P	*Rickettsia gltA*	[CY5]‐CATTGTGCCATCCAGCCTACGGT‐[BHQ3]	
CNeGroEL‐F	*Ca*. N. mikurensis *groEL*	CCTTGAAAATATAGCAAGATCAGGTAG	Jahfari et al., 2012
CNeGroEL‐R	*Ca*. N. mikurensis *groEL*	CCACCACGTAACTTATTTAGCACTAAAG	
CNeGroEL‐P	*Ca*. N. mikurensis *groEL*	[FAM]‐CCTCTACTAATTATTGCWGAAGATGTAGAAGGTGAAGC‐[BHQ1]	
ApMSP2F	*A. phagocytophilum Msp2*	ATGGAAGGTAGTGTTGGTTATGGTATT	Courtney et al., 2004
ApMSP2R	*A. phagocytophilum Msp2*	TTGGTCTTGAAGCGCTCGTA	
ApMSP2P	*A. phagocytophilum Msp2*	[CY5]‐TGGTGCCAGGGTTGAGCTTGAGATTG‐[BBQ650]	
Bab18S‐F	*Babesia* 18S rRNA	CAGCTTGACGGTAGGGTATTGG	Radzijevskaja et al., 2008
Bab18S‐R	*Babesia* 18S rRNA	TCGAACCCTAATTCCCCGTTA	
Bab18S‐P	*Babesia* 18S rRNA	[HEX]‐CGAGGCAGCAACGG‐[BHQ1]	
TBE1‐F	TBEV non‐struct. prot. 5	GGGCGGTTCTTGTTCTCC	Schwaiger & Cassinotti, 2003
TBE1‐R	TBEV non‐struct. prot. 5	ACACATCACCTCCTTGTCAGACT	
TBE‐Probe‐WT	TBEV non‐struct. prot. 5	[HEX]‐TGAGCCACCATCACCCAGACACA‐[BHQ2]	
FTu23‐F	*F. tularensis* 23Kda	TGAGATGATAACAAGACAACAGGTAAC	Skottman et al., 2007)
FTu23‐R	*F. tularensis* 23Kda	GGATGAGATCCTATACATGCAGTAGGA	
FTu23‐P2	*F. tularensis* 23Kda	[HEX]‐CCATTCATGTGAGAACTG‐[BHQ1]	
PCR:			
CS877f	*Rickettsia gltA*	GGGGACCTGCTCACGGCGG	Mediannikov et al., 2004
CS1258r	*Rickettsia gltA*	ATTGCAAAAAGTACAGTGAACA	
Bab‐F	*Babesia* 18S rRNA	GACACAGGGAGGTAGTGACAAG	Georges et al., 2001
Bab‐R	*Babesia* 18S rRNA	CTAAGAATTTCACCTCTGACAGT	
BbFLA‐F	*B. burgdorferi flagellin*	AGAGCAACTTACAGACGAAATTAAT	Skotarczak et al., 2002
BbFLA‐R	*B. burgdorferi flagellin*	CAAGTCTATTTTGGAAAGCACCTAA	

**Table A2:** Detected co‐infections in host‐seeking nymphs from Helsinki

	Bga	Bbss	Bva	Bmi	Bbsl	Rspp	Neo	Ana
Double infections								
Baf	**5** (0.4)	**7** (0.5)	**0**	**3** (0.2)	NA	**17** (1.2)	**12** (0.9)	**1** (0.07)
Bga		**1** (0.07)	**3** (0.2)	0	NA	**3** (0.2)	**1** (0.07)	**1** (0.07)
Bbss			**1** (0.07)	0	NA	0	0	0
Bva				0	NA	**2** (0.1)	0	0
Bmi					NA	**1** (0.07)	**1** (0.07)	0
Bbsl						**2** (0.1)	0	0
Rspp							**1** (0.07)	**1** (0.07)
Neo								0
Multiple infections								
Baf + Rspp+Cne	2 (0.10)							
Baf + Rspp+Bbss	2 (0.10)							
Baf + Bbss+Bga	1 (0.07)							
Bga + Bva+Rspp	1 (0.07)							
Baf + Rspp+Babe	1 (0.07)							

Abbreviations: Ana, Anaplasma phagocytophilum; Bga, *Borrelia garinii*; Baf, *B. afzelii*; Bva, *B. valaisiana*; Bbss, *B. burgdorferi* s.s.; Bmi, *B. miyamotoi*; Bbsl, *B. burgdorferi* s.l. (unidentified genospecies); Neo, Neoehrlichia mikurensis; Rspp, Rickettsia.

**Table A3:** Results from the statistical analyses regarding tick densities

Life stage	Fixed effect	Random effect	*N*	Ndf	Ddf	*F*	*p*
Models for study site‐specific tick densities							
Larvae	Study site	Month	3,115	7	3,103	15.12	<.0001
Nymphs	Study site	Month	3,115	7	3,103	38.36	<.0001
Adults	Study site	Month	3,115	7	3,103	13.61	<.0001
Models for study month‐specific tick densities							
Larvae	Month	Study site	3,115	4	3,103	20.99	<.0001
Nymphs	Month	Study site	3,115	4	3,103	58.36	<.0001
Adults	Month	Study site	3,115	4	3,103	13.49	<.0001

**Table A4:** Visitor count data sources

Study site(s)	Visitor count data source
Pähkinäinen, Vepsä, Maisaari	Pers. comm. Timo Sirkiä (Turku Urban Environment Division)
Boskär, Berghamn	Saaristomeren kansallispuiston kävijätutkimus 2014 Archipelago national park visitor survey 2014 (Metsähallituksen luonnosuojelujulkaisuja. Sarja B 223)
Seili, Ruissalo Botanical Garden	Pers. comm. Ilari Sääksjärvi (University of Turku, Biodiversity Unit)
Meilahti, Lauttasaari, Lehtisaari	Junttila et al. (1999)
Seurasaari	http://museovirastorestauroi.nba.fi/museot/seurasaaren‐ulkomuseo
